# Patient safety culture in primary and home care services

**DOI:** 10.1186/s12875-020-01263-1

**Published:** 2020-09-12

**Authors:** Letícia Martins Lousada, Francisco Clécio da Silva Dutra, Beatriz Viana da Silva, Natália Lúcia Lima de Oliveira, Ismael Brioso Bastos, Patrícia Freire de Vasconcelos, Rhanna Emanuela Fontenele Lima de Carvalho

**Affiliations:** 1 Ceara State University, Mister Hull avenue. 2933, apto 401b, violete, Fortaleza, Ceará Brazil; 2 University of the integration of Brazilian lusophony, Redenção, Ceará Brazil

**Keywords:** Patient safety culture, Primary care, Home care services

## Abstract

**Background:**

Safety culture is still a poorly studied subject in primary care and home care, although these settings are considered gateways to access to healthcare. This study aims to evaluate safety culture in primary and home care settings.

**Methods:**

An observational cross-sectional study was carried out with 147 professionals from nine districts covered by one home care program and six primary healthcare centres. The Safety Attitudes Questionnaire (SAQ) was used to evaluate the safety culture, in which scores ≥75 are indicative of a positive safety culture.

**Results:**

A total of 56 (86,1%) questionnaires returned from the home care professionals and 91 (86,6%) from the primary care professionals. The Job satisfaction domain was the best evaluated, achieving a score of 88.8 in home care and 75.1 in primary care. The achievement of high scores on Safety Climate, Job Satisfaction, Teamwork Climate, and Total SAQ was related to male gender, and time of professional experience of three to 4 years. Perception of management and Working conditions had the lowest scores, and this result was related with long time of experience.

**Conclusions:**

It is concluded that professionals working in home care gave higher scores for safety culture in their workplace than the primary care workers.

## Background

Providing safe care means changing attitudes and practices of all professionals involved in patient care. In the workplace, this requires a safety culture that strengthens the commitment and performance of the multidisciplinary team, as well as specific competencies to ensure patient safety [1].

According to the World Health Organization (WHO), patient safety is the reduction of risk of unnecessary harm associated with health care to an acceptable minimum. To ensure patient safety in Brazil, the National Patient Safety Program (PNSP in Portuguese) stands out promoting safety culture and emphasizing the importance of learning about patient safety and organizational improvement. This program reinforces that professionals must adhere to incident prevention and that institutions must develop safer systems and processes that avoid individual accountability for success or failure of care [2].

Safety culture is the product of a set of values, attitudes, perceptions, competencies, and behaviours that determine the commitment, style, and competence of an individual or a group in safety promotion. These behaviours include how managers and professionals act to improve healthcare, for example through collective learning and correction of errors [3]. However, it is observed that strategies developed for the implementation of a safety culture have not been extensively applied to primary and home care services [4, 5].

Patient safety is sometimes neglected in primary and home care centres. Actions and research in this theme are mostly conducted in hospitals and clinics since the culture of patient safety outside the hospital is still a challenge to be overcome [6, 7].

In Brazil, primary care is recognized as the gateway to the health system. Services provided in Brazilian primary care facilities directly affect the well-being of Brazilian families and the use of public resources. Consequently, insecure, inadequate, or ineffective primary care can increase preventable morbidity and mortality or lead to unnecessary use of hospital resources [8].

Home care services are part of a federal program that seeks to expand and qualify care within the Brazilian Unified Health System (SUS in Portuguese). The system is composed of services and actions that complement other levels of care, especially the tertiary and outpatient levels, ensuring the continuity of care and the integration of services within the network [9].

In principle, the SUS offers a patient-centred approach. Qualified professionals are assigned to geographical areas enabling the establishment of relationships between individuals and healthcare providers, in a way in which the professionals are familiar with the community’s routine, culture, and families. This approach favours the execution and articulation of actions such as rehabilitation, prevention, education, and health promotion. Thus, the Brazilian SUS is recognized as a system that favours patient safety movements and the implementation of a safety culture [10, 11].

The prioritization of patient safety in high complexity services led to a scarcity of studies on the subject in primary and home care settings, representing gaps in research and practices [12, 13]. Safety actions must be studied in the way in which they are practiced, and considering the perceptions of health workers to elucidate possible needs for improvements.

Instruments that assess patient safety are important tools to measure aspects such as organizational conditions that lead to incidents during health care, contributing to awareness of safety issues. This type of investigation helps to diagnose factors that influence the safety culture and patient safety [14, 15].

The Safety Attitudes Questionnaire (SAQ) is one of the tools created to provide a situational diagnosis of a service and/or institution, which enables an accurate assessment of factors that need to be improved and that influence safety, such as teamwork, job satisfaction, and working conditions [16].

Thus, the objective of this study was to evaluate safety culture in primary and home care settings and to verify relationships between the SAQ domains and variables related to gender, type of service (primary or home care), and time of professional experience.

## Methods

This is a cross-sectional study conducted in one home care service and in six primary care centres located in the metropolitan region of Fortaleza, Brazil. The data collection took place from January to July 2019.

The metropolitan region of Fortaleza in the state of Ceara consists of 19 cities, however, only nine are covered by the “Best at Home” program, namely: Cascavel, Eusebio, Guaiuba, Itaitinga, Horizonte, Maranguape, Maracanau, Pacatuba, and Sao Goncalo do Amarante. The six primary care centres included in the study are located in Fortaleza (one centre) and Acarape (five centres). These centres were chosen by convenience as they were accessible for the researchers involved, and due to willingness to participate.

All 69 professionals working in the Multiprofessional Home Care Team (MHCT) of the nine cities, and all 95 professionals working in the six primary care centres were invited to participate (intentional and non-probabilistic sample). Professionals developing their work activities during the data collection and with 6 months or more of time of experience were included. Professionals who were on leave or vacation were excluded.

The Safety Attitudes Questionnaire (SAQ) in its Brazilian translated and validated version was used for data collection [16]. The questionnaire was sent to the participants via Google Forms. The SAQ is one of the most used questionnaires to assess safety culture. Several studies present evidence of the validity [17, 18]and reliability [19, 20] of the SAQ in several languages [16–18, 21].

The SAQ is divided in two parts. The first part contains 41 items divided into six domains and the second part refers to professionals’ information. The domains are: Teamwork climate, Job satisfaction, Perception of unit and hospital management, Safety climate, Working conditions, and Perception of stress. The questionnaire items are presented in a 5-point Likert scale format. The final score of the SAQ ranges from zero to 100, in which zero corresponds to the worst perception and 100 to the best perception of safety culture, and scores ≥75 are indicative of a positive safety culture [22].

The collected data were tabulated in an Excel 2007® spreadsheet and analysed using the Statistical Package for Social Science (SPSS) version 22.0. To investigate the existence of differences between the mean scores of the SAQ domains, we used the analysis of variance (ANOVA) for quantitative variables and the Kruskal-Wallis test for qualitative variables, considering a significance level of *p* < 0.05.

Multiple regression analysis was performed for the adjustment of the predictive model. Dependent or response variables were defined considering the scores of each SAQ domain and the total SAQ value, for the following independent variables: gender, type of service (primary care or home care), and time of professional experience.

Before starting the research, consent from the managers of each study site was obtained. In agreement with the ethical and legal aspects, all participants were invited to participate in the research by signing an informed consent form. The study was approved by two Institutional Review Boards at the universities in which the project took place (protocols no. 2.943.854 and no. 2.522.957).

## Results

During data collection, a total of 65 questionnaires were distributed to the home care teams and 56 returned, while 105 questionnaires were distributed in the primary care centres and 91 returned. Four professionals were absent due to vacation or leave. In addition to these, six professionals did not return the questionnaire or refused to participate. This resulted in a rate of return of 86.1% from home care professionals and 86.6% from primary care professionals.

From the 147 participants, 98 (66.7%) were female and 91 (61.9%) were primary care workers, with up to 2 years of professional experience (58, 39.5%). Community Health Agents (CHA) represented 23 (15.6%) participants of the sample, followed by 22 (15%) nursing technicians and 20 (13.6%) physicians (Table 1).

**Table 1:** Profile of professionals participating in the research (*n* = 147)

Variables	n	%
**Gender**		
Female	98	66.7
Male	49	33.3
**Type of service**		
Home care	56	38.1
Primary care	91	61.9
**Profession**		
Community health agent	23	15.6
Nursing technician	22	15
Physician	20	13.6
Nurse	19	12.9
Physiotherapist	16	10.9
Administrative Support	12	7.5
Psychologist	10	6.8
Social worker	5	3.4
Speech therapist	4	2.7
Other^a^	16	2.0
**Time of professional experience**		
Less than 6 months	11	7.5
6 to 11 months	12	8.2
1 to 2 years	35	23.8
3 to 4 years	29	19,7
5 to 10 years	23	15.6
11 to 20 years	12	17
More than 20 years	12	8.2

^a^Nutritionists, pharmacists, occupational therapists, dentists

The total SAQ score was 68.5 (±14.4), indicating that the primary and home care services evaluated, in general, did not reach a positive value for the safety culture (cutoff value of 75). The scores of the SAQ domains ranged from 57.3 to 80.4. Job satisfaction obtained the highest value (80.4, ±15.8), which means that professionals were satisfied with their job; on the other hand, Working conditions and Management perception presented the lowest scores (Table 2).

**Table 2:** Safety Attitudes Questionnaire (SAQ) scores by domain

Domains	Mean	SD*	Median	Min	Max	75th percentile
Teamwork climate	75.8	21.5	79.1	25	100	87.5
Safety climate	68.6	15.8	83.3	0	100	90
Job satisfaction	80.4	15.8	83.3	25	100	94.8
Perception of stress	64.1	27.2	62.5	0	100	87.5
Management perception	57.9	23.5	60	0	100	75
Working conditions	57.3	27.8	58.3	0	100	75
Total SAQ	68.5	14.4	72.2	25	100	80.3

Correlations between gender, type of service (primary care versus home care), time of professional experience, and the SAQ domains were significant in regard to gender, as men gave higher scores for the domains Safety climate, Perception of stress, and Management perception than women. Home care professionals gave higher scores than primary care professionals for all domains, except Perception of stress. In addition, professionals working for 3 to 4 years tended to attribute high scores to the domains Safety climate, Job satisfaction, Teamwork climate and Total SAQ score (*p* < 0001). Men from the home care teams, with 3 to 4 years of professional experience evaluated safety culture in their workplace more positively (Table 3).

**Table 3:** Comparison of the averages of the SAQ domains and the variables gender, service, and time in service

	Domains						
	SC	JS	PS	TC	MP	WC	Total SAQ
**Gender**							
Female	65.2	79.8	57.6	74.5	52.6	54.5	65.7
Male	75.4	81.5	77	78.5	68.9	62.9	74
	**< 0.05**		**< 0.001**		**< 0.001**		**< 0.001**
**Type of service**							
Home care	83.4	88.8	61.4	86.3	63.8	67.1	78
Primary care	59.5	75.1	65.8	69.4	54.5	51.2	62.6
	**< 0.001**	**< 0.001**		**< 0.001**	**< 0.03**	**< 0.001**	**< 0.001**
**Time of professional experience**							
Less than 6 months	61.4	70.6	58.3	73.1	62.3	42.9	62.3
6 to 11 months	78.5	78.8	76	79.8	66.7	68	73.5
1 to 2 years	77.3	84.6	62.5	79.5	67.9	70.1	74.8
3 to 4 years	79.3	86.8	67.6	83.9	63.4	62.2	75.5
5 to 10 years	70.3	80.8	69.4	81.9	57.5	58	70.6
11 to 20 years	48,3	75.3	57.3	59	39	41.6	55.2
More than 20 years	53.6	72.9	57.8	67.9	45	42.3	57.6
	**< 0.00**	**< 0.002**		**< 0.00**	**< 0.00**	**< 0.001**	**< 0.00**

Safety Climate (SC), Job satisfaction (JS), Perception of stress (PS), Teamwork climate (TC), Management perception (MP), and Working conditions (WC)

The data presented in Table 4 reveal the values of standardized and non-standardized coefficients, in addition to t test, indicating how much the variables gender, type of service and time of professional experience influenced the SAQ domains (Table 4).

**Table 4:** Multiple linear regression of response and explanatory variables

Dependent variables	Independent variables	Non-standardized coefficients	Standardized coefficients	R^**2**^	t	p
Safety Climate	Gender	11.88	.26	.393	3.86	0.000
	Primary/home care	−23.76	−.54		−7.95	0.000
Job satisfaction	Time in service	−2.07	−.16		−2.35	0.002
	Primary/home care	−14.64	−.45	.174	−5.69	0.000
Perception of stress	Gender	19.10	.33	.096	3.14	0.001
Safety climate	Primary/home care	−16.52	−.47	.262	−6.32	0.000
Management perception	Gender	14.66	.29	.215	3.81	0.000
	Time in service	−3.81	−.27		−3.45	0.001
Working conditions	Primary/home care	−15.06	−.26	.107	−3.17	0.002
Total SAQ	Gender	9.60	.31	.392	4.64	0.000
	Primary/home care	−15.80	−.53		−7.87	0.000

The adjusted R2 values from the analyses ranged from 0.096 to 0.393. Adjusted R2 values suggest that 39% of the variation in Safety climate and total SAQ score can be explained by the variables gender, type of service, and time of experience. The R2 adjusted value for the domain Teamwork climate suggests that it accounts for 26% of the variation in the type of service (primary care versus home care).

## Discussion

Safety culture was evaluated by 147 healthcare professionals working in primary and home care services. Most of them were female and worked as community health agents, with time of professional experience of one to 2 years. In Brazil, community health agents have an important role in establishing “the link” between families and the primary care providers. Their work is essential for the successful approach of the families as it provides essential elements to the understanding of the families’ health problems and needs [23].

The total score obtained in the present study considering both types of service was below the cutoff value that classifies a positive safety culture. Besides that, there was a significant difference showing that home care workers gave higher scores of safety culture in their workplace than primary care workers. Although no studies have been found in the literature comparing the perceptions of primary and home care workers about safety culture, SAQ scores given by primary care workers in another study were close to ours [13].

A study conducted in a primary care setting at the southern region of Brazil found negative scores for safety culture in almost all SAQ domains [13]. An opposite result was identified in another study in which primary care providers and oral health workers evaluated safety culture using the Medical Office Survey on Patient Safety Culture (MOSPSC) questionnaire, and rated safety culture positively [12].

It was also found that Job satisfaction achieved the highest value, while Working conditions and Management perception had the lowest scores, with significant differences related to type of service and time of experience. These findings indicate that the participants do what “they like to do”. However, the low scores of management perception indicate that they do not approve (or partially approve) actions of their leaders regarding patient safety issues. These results agree with previous studies that used SAQ in Brazil and in other countries. A study conducted with professionals from five homes in Tonsberg, Norway, found high scores of Job satisfaction, followed by Teamwork climate and Safety climate [24]. In another study developed in home care services in Norway, Teamwork climate was the dimension with the highest score [25].

A Brazilian study also found that health professionals working in primary care have difficulty in working relationships with their managers and avoid commenting on work-related problems because they do not feel safe. In relation to this issue, managers recognized that communication problems are real in their workplace [8]. These situations can weaken patient safety in primary care.

In the present study, men who work in home care with three to 4 years of experience attributed high scores for Safety Climate, Job Satisfaction, Teamwork Climate, and the total SAQ. This means that these professionals enjoy the work that they do and have a positive view of the relationships that occur in their workplace. Until the completion of this study, no research was identified that justifies the difference in perceptions of safety culture between men and women. However, a study conducted in China with 2584 professionals identified that women gave higher scores than man to all SAQ domains, different from what was identified in our study [26].

Positive scores in the SAQ domains may indicate that professionals are satisfied with their own performance at work in situations where patient care may not be ideal. For this reason, managers should interpret the results with caution and consider the need for quality improvement interventions [24].

Regarding the type of service, a study identified a similar result when assessing safety culture in home care services [27]. In addition, according to a Brazilian study developed in the home care services, with users and providers, participants were satisfied with the health care program and this feeling would be the result of support provided by the family health teams, even in face of incidents [28].

Regarding time of professional experience, a study shows that professionals with long time of experience tend to be more critical regarding management actions and work environment characteristics [29], which may justify the lower scores of Working conditions and Management perception found in the present study among those with long time of professional experience. Management perception and Teamwork climate are domains that influence all the others SAQ domains, except Perception of stress [21].

The statistically significant correlations do not necessarily indicate an underlying relationship between the variables. Our analyses measured how much independent variables explains the response variable, through multiple linear regression. The regression model showed that gender, type of service, and time of professional experience contributed (positively and negatively) to Safety climate, Job satisfaction, Perception of stress, Teamwork climate, Management perception, Working conditions, and Total SAQ. Other studies have similar results and report, from multiple linear regression equations, that demographic factors such as gender, age, and participation in training significantly affect SAQ [26, 30].

## Limitations

This study has some limitations. First, self-reported surveys like the SAQ depend on the respondents’ recall and may be subject to reporting bias. However, the validity of the data gathered in the study is supported by their consistency and by the fact that the SAQ is widely used and has good psychometric qualities. Second, the cross-sectional approach limits our ability to establish assertions about change through time. Third, the convenience sample and the local level of the study limits the generalizability of the findings to other contexts.

In addition, there is a scarcity of studies evaluating safety culture in primary and home care settings, and the number of studies about the dimensions of SAQ and its relationship to demographic variables is small. We suggest that further studies are needed with a larger number of health units, in other regions of the country and in other countries, to determine which factors influence safety culture in different contexts. Such factors can be used in the formulation of public policies aimed at strengthening the safety culture in primary and home care settings.

## Conclusion

It is concluded that professionals working in home care gave higher scores for safety culture in their workplace than the primary care workers. In addition, men from the home care teams, with 3 to 4 years of professional experience evaluated safety culture in their workplace more positively and professionals with long time of experience were more critical regarding Working conditions and Management perception.

It should be noticed that this study comprehends perceptions, which are only a part of safety culture. Patient safety is not necessarily and solely associated with high quality diagnoses, adequate treatment, and patient-centred care. To further explore this subject, it is necessary to investigate associations between safety culture and the occurrence of incidents in settings like the ones investigated in this study.

Finally, as an implication of this study, the findings can motivate managers to promote the safety culture in the participant settings and can increase professional awareness of the factors that influence safety culture and consequently patient safety.

## Data Availability

The study database is available by contacting the corresponding author.

## Abbreviations

WHO: World Health Organization
PNSP: National Patient Safety Program
SAQ: Safety Attitudes Questionnaire
MHCT: Multiprofessional Home Care Team
SPSS: Statistical Package for Social Science
ANOVA: Analysis of variance
CHA: Community Health Agents
MOSPSC: Medical Office Survey on Patient Safety Culture
